# Uniform dipole resonance and suppressed quadrupole resonance for constant transmittivity full phase control plasmonic metasurfaces

**DOI:** 10.1038/s41598-024-83191-z

**Published:** 2024-12-28

**Authors:** Subrata Karmakar, Anil Ringne, Nirjhar Kumar, Ananth Krishnan

**Affiliations:** Department of Electrical Engineering, Centre for NEMS and Nanophotonics (CNNP), Indian Institute of Technology Madras, Chennai, 600036 India

**Keywords:** Metamaterials, Nanoparticles, Nanophotonics and plasmonics, Optical manipulation and tweezers

## Abstract

Transmission-type plasmonic phase metasurfaces utilizing the Pancharatnam-Berry (PB) phase require constant transmittivity with complete phase variation from 0 to 2$\pi$. Usually, this is achieved by rotating metallic nanoparticles in an otherwise uniform lattice arrangement. However, this rotation and the chosen lattice structure cause a significant change in the transmittivity, resulting in a lower intensity of light with certain phases and a higher intensity for other phases. Even though they are called full phase metasurfaces, their intensities can be near maximum or near minimum depending on the rotation and the lattice structure. We show that it is possible to achieve full phase constant transmittivity metasurfaces using the PB phase and the most elementary metallic anisotropic nanoparticles (elliptical) by inserting a thin metal sheet between the nanoparticles and the substrate. Without this metal sheet, while full phase control could be achieved by merely rotating the particles, the transmittivity varies by about 50% depending on the nanoparticles’ orientation. With the metal sheet, full phase control from 0-2$\pi$ with a transmittivity variation of only 13%, even in a square lattice, is demonstrated with simulations and experiments. We show that this is due to the annihilation of quadrupole resonances along with broader uniform dipole resonance in the case of the nanoparticles with the metal sheet below. We also show that precise phase control is possible by generating varieties of orbital angular momentum beams and complex beams in the visible spectrum using nanofabricated metasurfaces.

## Introduction

Phase Metasurfaces (PM) allow manipulation of the phase of incident light using subwavelength structures. They find extensive applications in advanced imaging^1–3^, vortex generation^4–7^, thin waveplates^8–11^, holography^12–17^, etc. PMs can be engineered by adjusting Mie scattering with dielectrics^18^ or by utilizing the Pancharatnam-Berry (PB) phase utilizing either metals or dielectrics^19,20^, in transmission or reflection mode^21,22^. In the case of plasmonic metasurfaces, every nanoparticle must be asymmetric and show a rotation-dependent phase change to obtain full phase control. Most plasmonic PMs in the visible and infrared spectra utilize anisotropic nanoslits^21,23–26^, while some others utilize multilayered unit cells^27^. While phase control is demonstrated in these structures, the transmittivity changes arising due to the unit cell and lattice have pronounced effects, resulting in varying intensities of different phases, leading to aberrations at the output. Lattice induced transmittivity changes for varying phase controls are well pronounced in square lattice based PM^2,15,17^. To overcome this problem, alternate lattice arrangements can immediately be conceived as a solution^28^. However, it is worth noting that not all symmetric lattice configurations are possible due to the small spacing between the nanoparticles compared to their size. Hence, a full-phase plasmonic metasurface using the Pancharatnam-Berry Phase independent of lattice configuration is important. Metal nanoparticles over metal substrates^29^ have garnered interest in handling lattice induced transmittivity changes, as it is a novel method to engineer the nearfield of plasmonic nanostructures. In this work, we show that it is possible to create such a metasurface with a regular square lattice structure itself. We show that rather than adjusting lattice unit vectors, a thin metal film deposited before the lithographic definition of the elliptical nanoparticles can achieve full phase control with nearly constant transmittivity. We show using simulations that in the case of a square lattice arrangement of elliptical nanoparticles, both dipole and quadrupole resonances occur with different complementary contributions with the rotation of these particles, meaning that for specific rotations, the dipole contribution is less, and for the same case, the quadrupole contribution is more and vice versa. This results in a transmittivity variation of nearly 50%, thus rendering the usage of such metasurfaces to a limited scope. Using simulations, we also show that by merely depositing the thin uniform metal film before the lithographic definition of the nanoparticles, we suppress the quadrupole resonances, and we also show that the angular dependence of transmittivity due to the dipole resonance mode can be suppressed by broadening the resonance, resulting in uniform angle independent transmittivity. This results in a full phase control with negligible transmittivity variation in our proposed metasurface. We further nanofabricate the proposed metasurface on a glass substrate, with gold as the thin film and gold nanoparticles lithographically defined on top. To demonstrate the finesse with which we can control the phase, we experimentally demonstrate multiple composite vortex beams constituted with multiple topological charges. Further, in our structure, we show that the resultant field distribution is concentrated on the top corners of the nanoparticles. This has potential applications in sensing, as the field concentrated on the top of the nanoparticles can be perturbed by surface refractive index changes.

## Design

**Figure 1:**
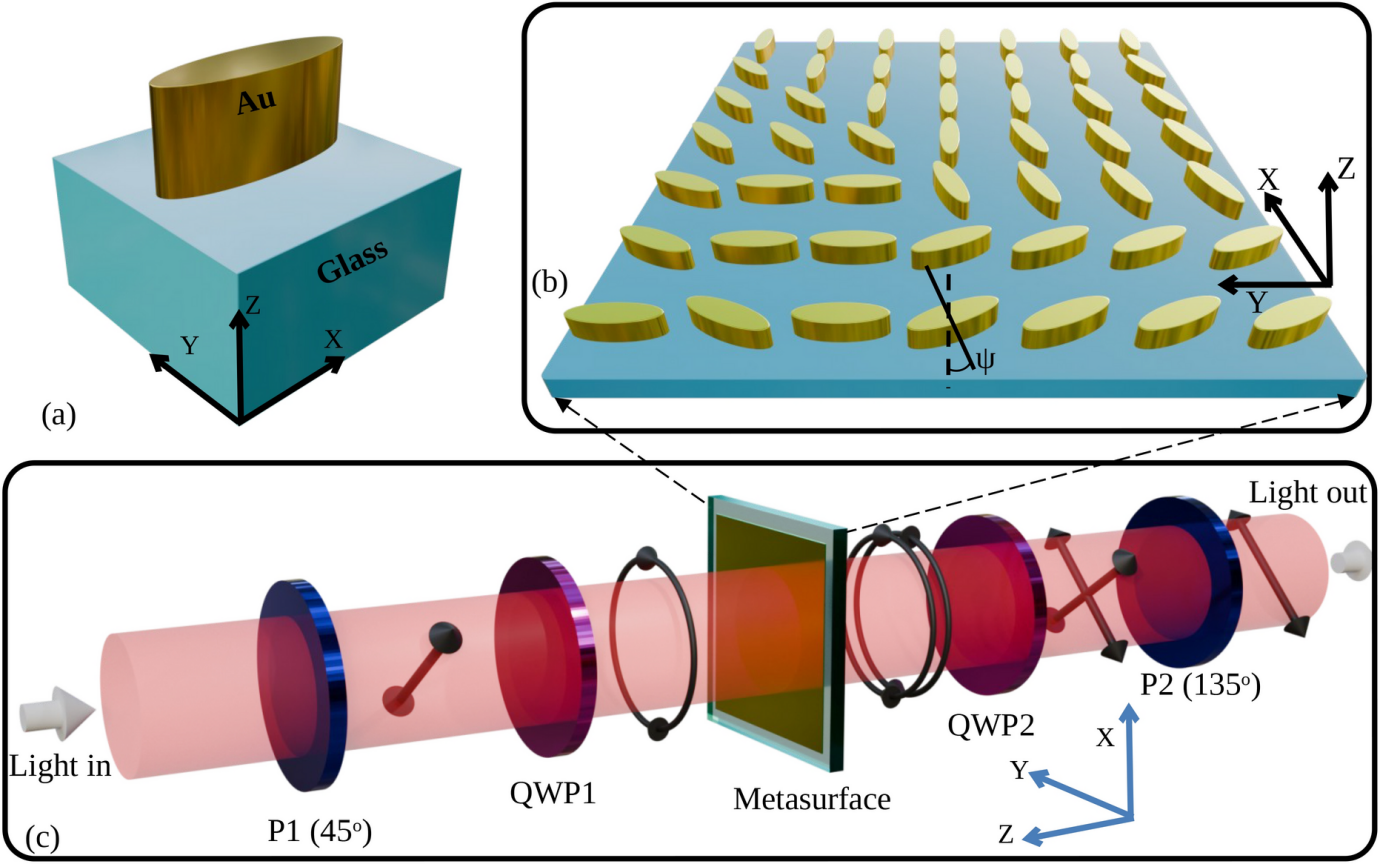
(**a**) Unit cell composed of a Metallic elliptical nanoparticle over a glass Substrate (MoS), (**b**) phase metasurface made of MoS unit cells arranged with different polarization angle $\psi$, (**c**) block diagram of the experimental setup. ‘P’ stands for polarizer, and ‘QWP’ stands for quarter wave plate.

Figure 1a shows the schematic (all the 3D images are created using Blender 4.0, https://www.blender.org and LibreOffice 7.0.4.2, https://www.libreoffice.org) of a metasurface unit cell composed of a Metallic elliptical nanoparticle over a glass Substrate (MoS). The experimental setup is shown in Fig. 1c. The electric field ($\hbox {E}_{QWP1}$) after the first polarizer (P1) and Quarter Wave Plate (QWP1), which is incident on the elliptical nanoparticles, can be written to be proportional to the Jones matrix of the Left Circularly Polarized (LCP) light, as shown by Eq. (1).

1 $$\begin{aligned} E_{QWP1}\propto \begin{pmatrix} 1 \\ j \end{pmatrix} \end{aligned}$$

When this circularly polarized light is incident from the top of the unit cell in Fig. 1a, in the negative Z direction, the geometrical anisotropy of the nanoparticle produces a differential phase shift between the major and minor axes of the elliptical nanoparticle. In other words, the elliptical nanoparticle acts as a nanopolarizer. The transmitted light through the substrate will ideally be linearly polarized, with the fast axis being the ellipse’s minor axis. This linearly polarized output can be represented as a sum of a right circularly polarized and a left circularly polarized light with equal amplitudes. However, in reality, the transmittivity along the major and minor axes is different. If the complex transmission coefficient along the major axis is represented by $t_0$ and that along the minor axis is $t_1$, then the transmitted electric field^30^ after the nanoparticle can be represented by Eq. (2).

2 $$\begin{aligned} \begin{aligned}&E_{meta}(x,y)=\frac{1}{2}(t_1 +t_0)\begin{pmatrix} 1 \\ j \end{pmatrix}+\frac{1}{2}e^{j2\psi (x, y)}(t_1 -t_0)\begin{pmatrix} 1 \\ -j \end{pmatrix} \end{aligned} \end{aligned}$$

Where *x* and *y* are the coordinates of each nanopolarizer with the orientation angle $\psi$ with respect to the axis. By using a combination of a quarter wave plate (QWP2) and a linear polarizer (P2), as shown in Fig. 1c, one of the circularly polarized light components can be allowed to pass through. Different elliptical nanoparticles placed in some lattice arrangement can each be rotated individually about their centroids in the XY plane as shown in Fig. 1b to produce circularly polarized light with different initial phases. The linear polarizer (P2) selects the polarization at the output. The output electric field is given by Eq. (3). Ideally, $t_0$ and $t_1$ should be real with values of 0 and 1, respectively, leading to constant transmittivity for different $\psi$. However, in practical scenarios, $t_0$ and $t_1$ are complex, with the amplitude of $a_0$ being greater than 0 with a phase $\xi /2$ and the amplitude of $a_1$ being less than 1 with a phase of $-\xi /2$.

3 $$\begin{aligned} E_{out}(x,y)=\frac{1}{2}e^{j2\psi (x,y)}(a_{1}e^{j\xi /2}-a_{0}e^{-j\xi /2})e^{j\frac{\pi }{4}}\begin{pmatrix} 1\\ -1 \end{pmatrix} \end{aligned}$$

In the above expression, $e^{j2\psi (x,y)}$ indicates that the phase of the electric field can be retarded locally by changing $\psi$, which can be adjusted from 0 to $\pi$. Hence, a total 2$\pi$ phase retardation is possible using described configuration. The orientations of P1, P2, and the fast axes of QWP1 and QWP2 are $45^\circ$, $-45^\circ$, $90^\circ$, and $0^\circ$ respectively, for the above calculation. In the case of partial polarizers, either linear or circular, the Jones matrix will be a Hermitian^31^, which implies that $\xi$ will be four times multiple of $\psi$. In addition, if $a_1$ and $a_0$ are nearly identical, transmittivity will depend sinusoidally on $\psi$. Therefore, a variation in transmittivity with respect to $\psi$ is expected. To obtain constant transmittivity with respect to $\psi$, the criterion should be that the difference between $t_1$ and $t_0$ should be maximum and real. The design of such a unit cell that satisfies the above criterion is not trivial. Hence, further simulation is performed to analyze the transmittivity and phase characteristics of the MoS and the proposed unit cell that can satisfy this criterion.

**Figure 2:**
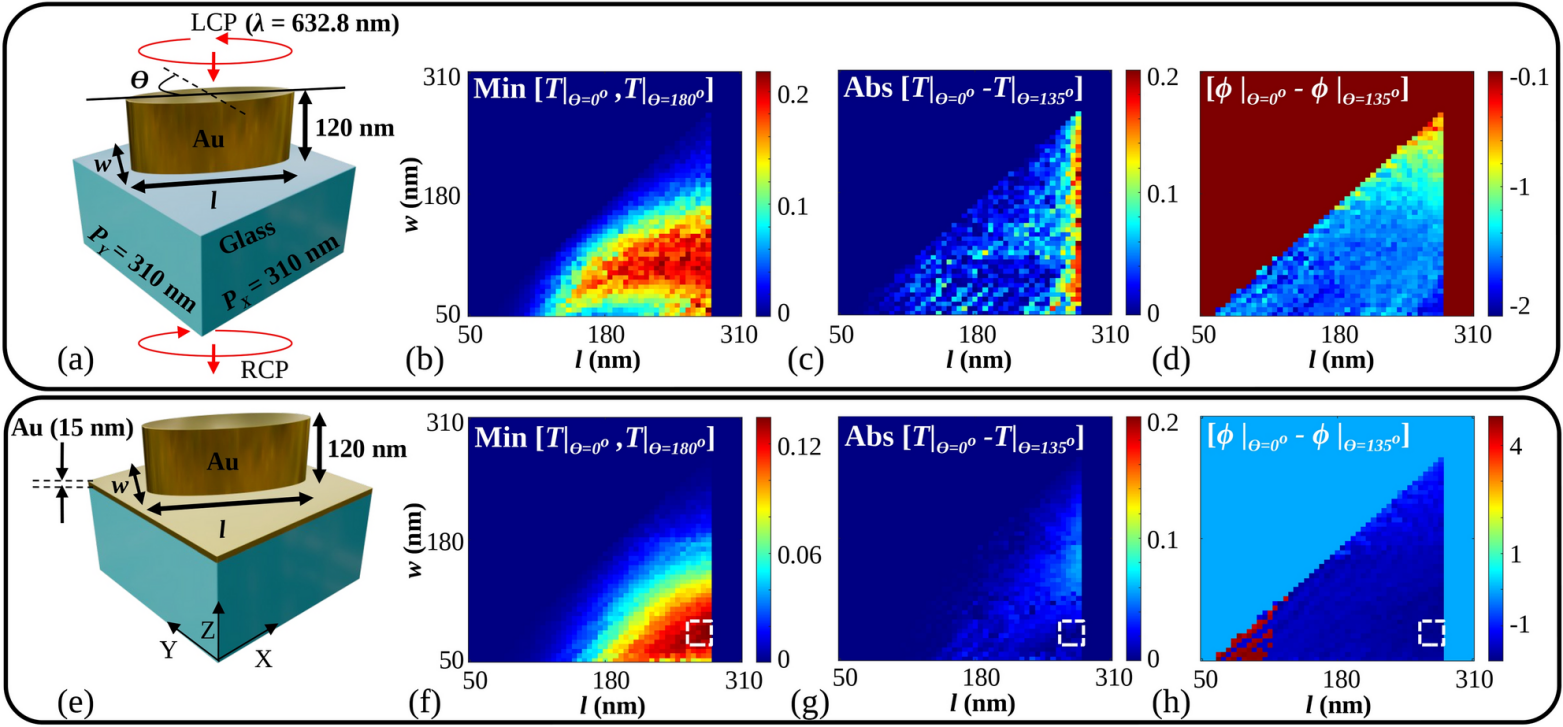
(**a**) Cross section of the MoS unit cell, (**b**) minimum value between the transmittivities, T for $\theta =0^\circ$ and $\theta =135^\circ$ for MoS unit cell, (**c**) difference between the transmittivities, T at $\theta =0^\circ$ and $\theta =135^\circ$ for MoS unit cell, (**d**) difference between the phase, $\phi$ for $\theta =0^\circ$ and $\theta =135^\circ$ for MoS unit cell, (**e**) proposed cross section of a unit cell composed of Metallic elliptical nanoparticle over metal (MoM) sheet on a glass substrate, (**f**) minimum value between the transmittivity, T for $\theta =0^\circ$ and $\theta =135^\circ$ for the MoM unit cell, (**g**) difference between the transmittivities, T at $\theta =0^\circ$ and $\theta =135^\circ$ for the MoM unit cell, (**h**) difference between the phase, $\phi$ for $\theta =0^\circ$ and $\theta =135^\circ$ for the MoM unit cell. $\theta$ is the orientation angle of the major axis of the elliptical nanoparticle with respect to the Y axis. Input is taken as Left Circularly Polarized (LCP) light, and output is taken as Right Circularly Polarized (RCP) light. ‘Min’ and ‘Abs’ are abbreviated for minimum and absolute values.

The functional requirement of our unit cell is to work as a nanopolarizer, which necessitates the use of an asymmetric nanoparticle based unit cell. The most fundamental nanoparticle of the unit cell could be nanocuboids and nanoellipses due to their well-defined long and short axes. However, nanocuboids have sharp edges that get rounded due to fabrication inaccuracies. Therefore, we opted for nanoellipse as a practical alternative. In addition, the size of the unit cell is decided by the simulations to have maximum transmittivity and low transmission variations, with an additional constraint on the sizes being compatible with the tolerances of the electron beam lithography process. While full phase control is possible, it is important to establish the amplitude characteristics of the transmitted wave due to the varying transmittivities in different orientations of the elliptical nanoparticle. We perform this analysis using simulations. Figure 2a shows a cross-section of a glass substrate with an elliptical nanoparticle made of gold (Au) on the top. Fullwave Finite Difference Time Domain (FDTD) simulations using the Ansys Lumerical FDTD tool (Ansys Optics 2022 R2.4, https://www.ansys.com/en-in/products/optics/fdtd) were performed to calculate the transmittivity characteristics for LCP input and Right Circularly Polarized (RCP) output light. Since the simulated transmittivity is found to be the maximum for a certain period, the periods ($P_X$ and $P_Y$) of this unit cell along both the X and Y axes are fixed at 310 nm. The Fig. 2b shows the minimum value between the transmittivity for $\theta =0^\circ$ and $\theta =135^\circ$. In Fig. 2b, f, the minimum value is defined as the minimum transmittivity among the transmittivities obtained for the range of orientation angles of the unit cell from $0^\circ$ to $180^\circ$. These $\theta$ values are chosen according to the treatment of these nanoparticles as nanopolarizers, following Malus’s law for linearly polarized input light. From Fig. 2b, it is clear that depending on the choice of *l* and *w*, there is a significant change in the minima of transmittivity between $\theta =0^\circ$ and $\theta =135^\circ$. It is also clear that higher anisotropy increases the transmittivity, as evidenced by the red regions of Fig. 2b.

For maximum transmittivity, one has to choose the highest value of this minimum shown as a deep red color according to the color scale in Fig. 2b. To obtain maximum transmittivity along with the highest phase retardation efficiency, the fill factor of the nanoparticles needs to be high. This is clearly evidenced in Fig. 2b, where dense red regions are to the right of the plot. Figure 2c represents difference between the transmittivities at $\theta =0^\circ$ and $\theta =135^\circ$. For a constant transmittivity metasurface, we desire negligible difference between the transmittivities. In Fig. 2c, it is clear that the difference is significant if one chooses to maximize the transmittivity from Fig. 2b by choosing a value of *l* and *w*, in order to have high fill factor (note that the values plotted are absolute values of transmittivity and later in the manuscript, we show how in terms of percentage, this is a significant amount). The fill factor is defined as the ratio of the top surface area of the nanoparticle and the top surface area of the unit cell. From Fig. 2d, it is desired that the values of *l* and *w* be such that the difference between the phase for $\theta =0^\circ$ and $\theta =135^\circ$ be $-\pi /2$. From the combination of these figures, it is difficult to choose the values of *l* and *w* for the highest fill factor, with high transmittivity, with low variation in transmittivity, and a constant phase difference. The region in the subplots of Fig. 2, when *l* approaches 310 nm, appears uniform because simulations were not performed in this region. This decision was made because, at this length, the elliptical nanoparticle’s dimension approaches the period of the structure. If the period and the length of the elliptical nanoparticle are nearly the same, then the adjacent particles will touch each other. $\psi$ and $\theta$ are defined to be ninety degrees apart from each other.

**Table 1:** Optimized parameters of the MoM unit cell.

Parameters	Dimensions (nm)
$P_{X}, P_{Y}$	310
*l*	280
*w*	80
Height	120
Gold sheet thickness	5, 15

To solve the problem of angle dependent transmittivity, we propose a thin Au sheet of 15 nm be inserted between an elliptical nanoparticle and substrate, which has the cross section of a Metallic elliptical nanoparticle over a Metallic sheet (MoM) on a glass substrate, as shown in Fig. 2e. Keeping the height and the period fixed at 120 nm and 310 nm respectively, transmittivity characteristics are calculated using the same FDTD tool. Figure 2f shows the minimum value between the transmittivity of the MoM unit cell for $\theta =0^\circ$ and $\theta =135^\circ$. From Fig. 2f, it is clear that maximum transmittivity is obtained from the right side of the plot as deep red color is visible. Depending on the choice of *l* and *w* in this region, a high anisotropy of the elliptical nanoparticle can also be observed. Figure 2g shows the difference between the transmittivities of the MoM unit cell for $\theta =0^\circ$ and $\theta =135^\circ$ with various *l* and *w* values. It is shown by choosing the values of *l* and *w* in the region indicated with a white dotted box in Fig. 2f–h, one can obtain constant transmittivity. For the indicated region, where maximum transmittivity is observed, the difference between the transmittivities is also reduced, as shown in Fig. 2g. As desired, from Fig. 2h, for the indicated region, the difference between the phase for $\theta =0^\circ$ and $\theta =135^\circ$ is $-\pi /2$. As per the analysis from Fig. 2, the values of *l* and *w* are chosen such that a full phase metasurface with maximum transmittivity and negligible transmittivity difference can be obtained and shown in Table 1. The elliptical nanoparticles with chosen *l* and *w* also satisfy high fill factor and anisotropy.

**Figure 3:**
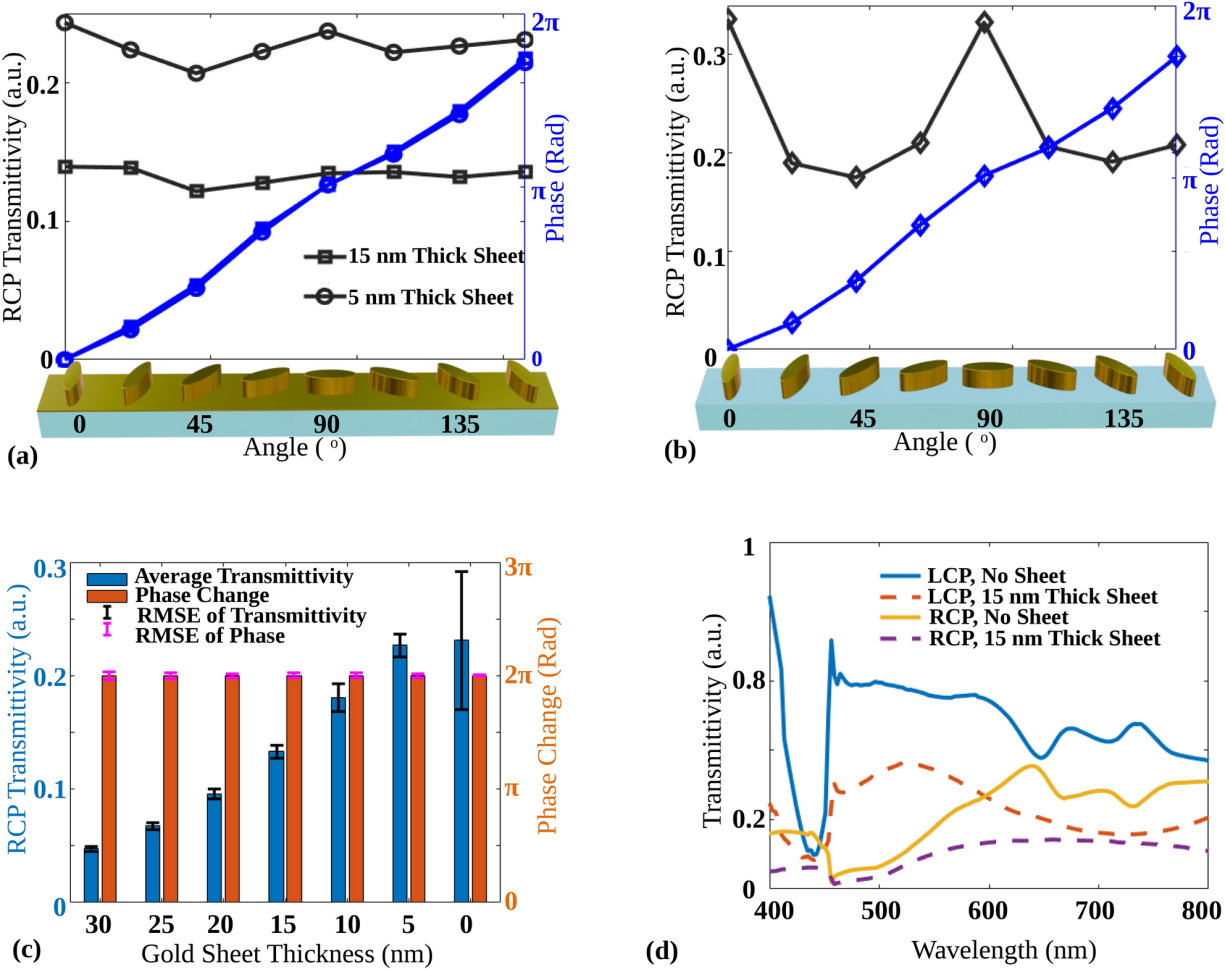
RCP transmittivity and phase plot for optimized parameters mentioned in Table 1 of (**a**) the MoM unit cell with elliptical nanoparticle thickness of 120 nm with two sheet thicknesses below them of 5 nm and 15 nm. (**b**) The MoS unit cell for different orientation angles. (**c**) Average transmittivity, the root mean square error (RMSE) of the transmittivity, phase change, and the RMSE of the phase change for different Au sheet thicknesses of MoM unit cell. For an Au sheet thickness of 0 nm, the MoS unit cell is considered, and (**d**) the RCP and LCP spectral response of the MoM and the MoS unit cell both for $\theta =0^o$.

To calculate the transmittivity and phase response for different orientation angles from 0 to $\pi$, optimized parameters mentioned in Table 1 are considered for further simulations. Figure 3a, b show the plots of transmittivity and phase of the MoM and MoS unit cells respectively, for RCP output light with different orientation angles. The orientation angles are considered as $0^\circ , 22.5^\circ , 45^\circ , 67.5^\circ , 90^\circ , 112.5^\circ , 135^\circ$ and $157.5^\circ$. For all the mentioned angles, the full $2\pi$ phase retardation is obtained for both unit cells. The MoS unit cell shows a transmittivity variation of about 47.92%, whereas the MoM unit cell has a variation of only 12.74% for a mere 15 nm sheet inserted below. The variation is calculated as the ratio of the difference between maximum and minimum transmittivities to the maximum transmittivity. From Fig. 3a, b, the effect of the metallic sheet, placed between the elliptical nanoparticle and substrate, on the transmittivity is clearly visible. To analyze the effect of the thickness of the metallic sheet on the transmittivity and the phase, Fig. 3c shows the calculated average transmittivity, the root mean square error (RMSE) of the transmittivity, phase change and the RMSE of the phase change. The average transmittivity is calculated by taking the mean of the transmittivities for mentioned orientation angles for the fixed height of the elliptical nanoparticle and the fixed thickness of the Au sheet. The RMSE of the transmittivity is calculated as root means square against average transmittivity, and the RMSE of the phase change is calculated against the linear increase in phase from 0 to 2$\pi$ for the fixed height of elliptical nanoparticle and fixed thickness of Au sheet. For the same conditions, the phase change is calculated as the difference between the maximum and minimum phase for the mentioned angles. As shown in Fig. 3c, for the thickness of Au sheet as 0 nm, high RMSE is observed along with high average transmittivity, which can be predicted from Fig. 3b.

**Figure 4:**
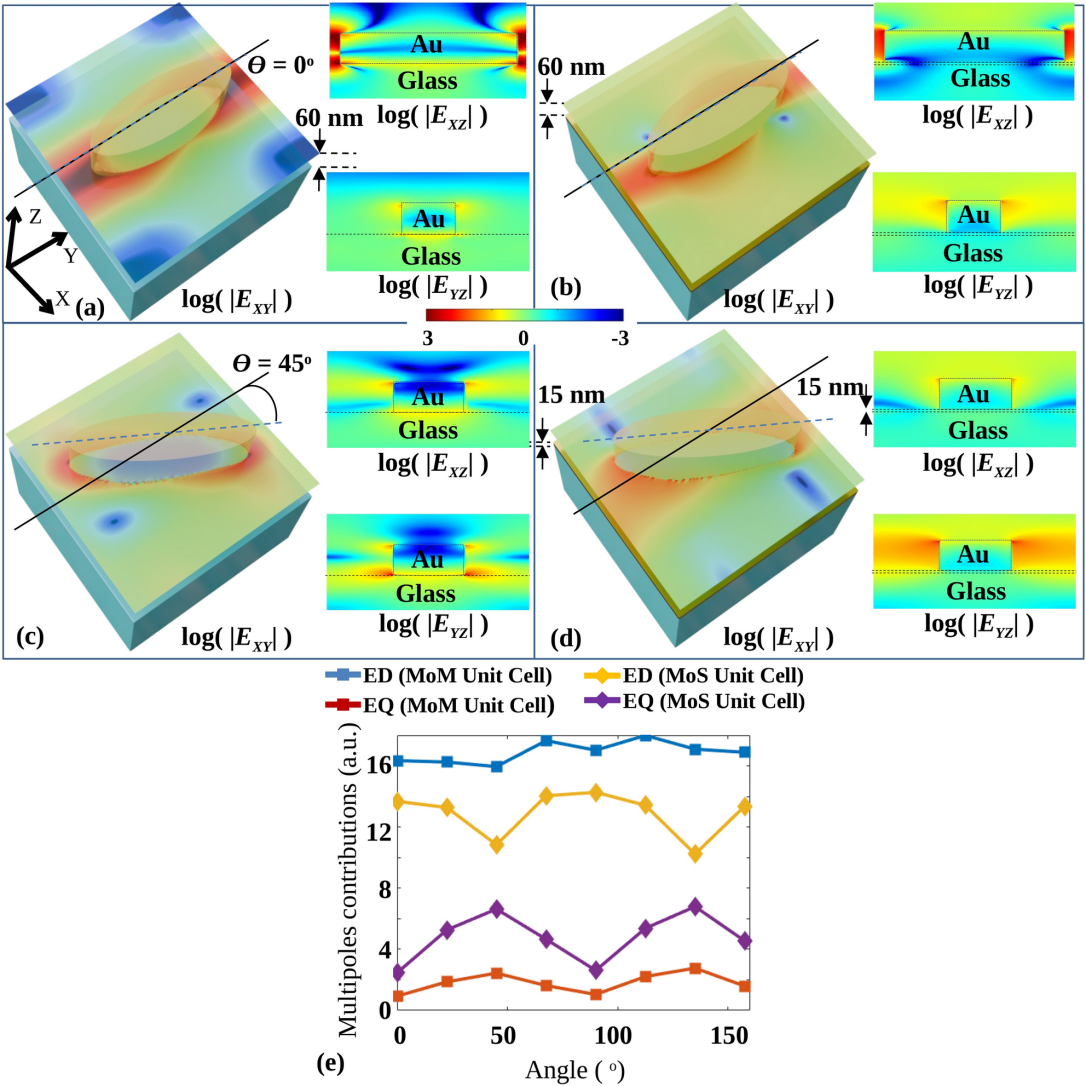
RCP Electric fields amplitude profiles $E_{XY}$, $E_{XZ}$ and $E_{YZ}$ for $\theta = 0^\circ$ orientation for (**a**) the MoS and (**b**) the MoM unit cell, electric fields amplitude profiles $E_{XY}$, $E_{XZ}$ and $E_{YZ}$ for $\theta = 45^\circ$ orientation for the (**c**) MoS and (**d**) the MoM unit cell, (**e**) absolute multipole contributions of electric dipole, ED and electric quadruple, EQ moments for MoS and MoM unit cells for different orientation angles.

In the case of a 5 nm Au sheet, an average transmittivity of 22.66% is obtained, decreasing further with the increase in Au sheet thickness. The average transmittivity of 13.31% is obtained for 15 nm sheet thickness. In all the cases, the phase change of total $2\pi$ is observed with minimum RMSE. As demonstrated in Fig. 3a–c, the insertion of a 5 nm thick Au sheet significantly contributes to maintaining a consistent transmittivity without notably reducing the average transmittivity. Specifically, Fig. 3c shows that the average transmittivity for an Au sheet thickness of 0 nm and 5 nm remains approximately the same. However, the variation in transmittivity is reduced when a 5 nm thick Au sheet is used, leading to a flatter transmittivity profile. If the thickness of a thin Au sheet further increases, the transmittivity variation decreases; moreover, the average transmittivity also decreases. Hence, there is a trade-off between high average transmittivity and low transmittivity variation. To analyze the spectral characteristics, the transmittivity of RCP and LCP are plotted and shown in Fig. 3d for the MoM unit cell as well as for the MoS unit cell with $\theta = 0^\circ$ orientation angle. RCP transmittivity of the MoM unit cell shows a broadened wavelength response compared to the MoS unit cell.

To analyze the transmittivity difference, the RCP electric field amplitude profiles are drawn for $\theta = 0^\circ$ and $\theta = 45^\circ$ as shown in Fig. 4. Figure 4a, b show logarithmic absolute electric fields $E_{XY}$, $E_{XZ}$ and $E_{YZ}$ for $\theta = 0^\circ$ oriented MoS and MoM unit cell, respectively. By inserting the metallic sheet between the elliptical nanoparticle and substrate, the electric field $E_{XY}$ is observed to be reduced. Electric field profiles $E_{XZ}$ and $E_{YZ}$ suggest the redistribution of the electric field from the substrate toward the top corners. Figure 4c, d show the logarithmic absolute electric fields $E_{XY}$, $E_{XZ}$ and $E_{YZ}$ for $\theta = 45^\circ$ oriented MoS and MoM unit cell, respectively. In this case, also, from the electric field profiles $E_{XZ}$ and $E_{YZ}$, the fields are observed to be redistributed from the substrate toward the top corners of the elliptical nanoparticle. For the MoM unit cell, the distribution of the electric field is nearly the same after rotation, whereas the distribution is changed for the MoS unit cell.

To analyze the relative contributions of ED and EQ, the moments of ED and EQ components of the transmittivity are calculated using the exact multi-pole expansion method^32^ for all the mentioned orientation angles for both the unit cells. For the LCP source, the electric field ${\varvec{E}}({\varvec{r}} )$ in X, Y, and Z directions are simulated using the FDTD tool, where *r* = (*x*, *y*, *z*) is the position vector. The angular frequency ($\omega$) and relative permittivity ($\varepsilon _d$) of the elliptical nanoparticle are also exported along with ${\varvec{E}}({\varvec{r}} )$ from FDTD simulation for calculating displacement current distribution (*J*(*r*)) of the unit cell. The *J*(*r*) of the unit cell^32^ can be obtained from Eq. (4).

4 $$\begin{aligned} \begin{aligned}&{\varvec{J}}({\varvec{r}} )={-} i\omega {\varepsilon _0}({{\varepsilon _d} - 1} ){\varvec{E}}({\varvec{r}} ) \end{aligned} \end{aligned}$$

where, $\omega$ is angular frequency, $\varepsilon _d$ is the relative permittivity of the elliptical nanoparticle, and $\varepsilon _0$ is a dielectric constant of vacuum. ED and EQ moments^32^ are obtained from the calculated *J*(*r*), as per the Eq. (5).

5 $$\begin{aligned} \begin{aligned}&\text {ED} = \sum _{i} \left|{-} \frac{1}{{i\omega }}\left[ {\int {J_i }{j_0}({kr} ){\text {d}^3}{\varvec{r}} + \frac{{{k^2}}}{2}\int \{{3({{\varvec{r}} \cdot {\varvec{J}}} ){r_i } - {r^2}{J_i }} \}\frac{{{j_2}({kr} )}}{{{{({kr} )}^2}}}{\text {d}^3}{\varvec{r}}} \right] \right|^2\\&\text {EQ}=\sum _{ij}\left|{-} \frac{1}{{i60\omega }}\left[ {\int \{{3({{r_j }{J_i } + {r_i }{J_j }} )- 2({{\varvec{r}} \cdot {\varvec{J}}} ){\delta _{i j }}} \}\frac{{{j_1}({kr} )}}{{kr}}{\text {d}^3}{\varvec{r}}} \right. + 2{k^2}\int \{{5{r_i }{r_j }({{\varvec{r}} \cdot {\varvec{J}}} )- {r^2}({{r_i }{J_j } + {r_j }{J_i }} )} \left. { { - {r^2}({{\varvec{r}} \cdot {\varvec{J}}} ){\delta _{i j }}} \}\frac{{{j_3}({kr} )}}{{{{({kr} )}^3}}}{\text {d}^3}{\varvec{r}}} \right] \right|^2 \end{aligned} \end{aligned}$$

Where *k* is the wavenumber and *i*, *j* = *x*, *y*, and *z*. The spherical Bessel function $j(\nu )$ is denoted by $j(\nu )=\sqrt{\pi /2\nu } J_{n+1/2}(\nu )$, where the Bessel function of first type is denoted by $J_n(\nu )$. The obtained ED and EQ moments are plotted in Fig. 4e. Figure 4e shows the absolute multipole contributions of ED and EQ moments. Significant variations in ED and EQ are observed, with complementary variations in each for MoS unit cells. It can be observed that ED is enhanced and nearly constant for MoM unit cells. For the same unit cell, EQ is quenched and shows negligible variation.

**Figure 5:**
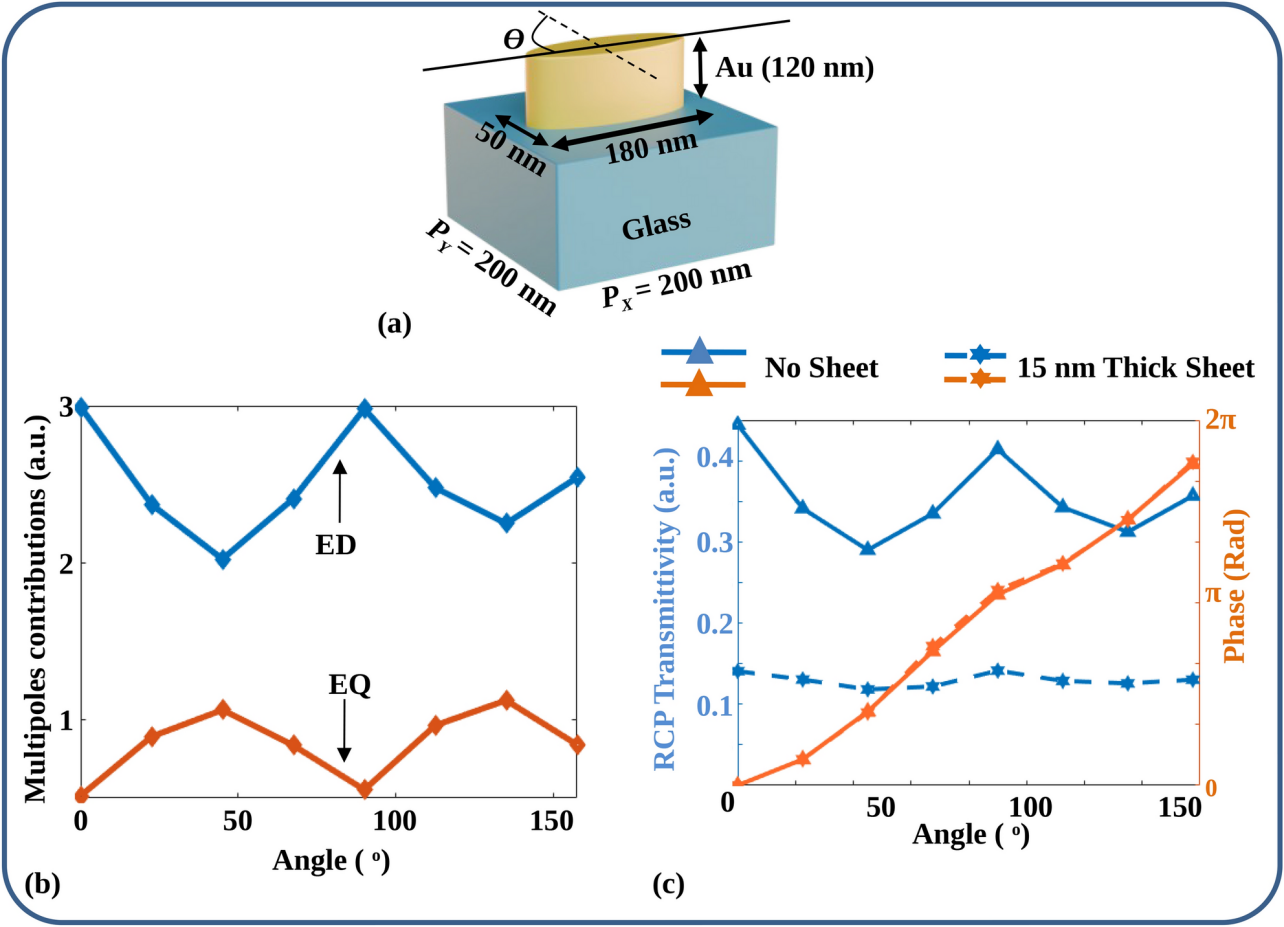
(**a**) Sketch of MoS unit cell with reduced dimensions and identical fill factor as the optimized MoM unit cell, (**b**) multipole contributions of the unit cell illustrated in (**a**), (**c**) simulated RCP transmittivity and phase for the MoM and MoS unit cells depicted in (**a**), maintaining reduced dimensions and the same fill factor as the optimized unit cell.

At this point, a valid question arises with regard to the possibility of suppressing the quadrupole moment by merely reducing the unit cell dimensions. This way, the size of the nanoparticle can be reduced such that the quadrupole mode is not supported. A natural question, in that case, could be whether a small unit cell would necessarily work as a nanopolarizer with uniform transmittivity at all angles. In the following figure, we show that this is not the case. Figure 5a shows the simulated Metal nanoparticle over a glass Substrate (MoS) unit cell with a length and width of 180 nm and 50 nm, respectively. The period is 200 nm to maintain the same fill factor in Table 1. The reason for picking 200 nm as the period is because, in this choice, the separation between two adjacent elliptical nanoparticles becomes 20 nm, which is normally the tolerance limit of the Electron beam lithography system. Figure 5b shows the corresponding multipole contribution to the transmittivity. Clearly, the EQ contribution is lowered. However, significant variations in both the ED and EQ are observed, with complementary variations as also observed in Fig. 4e. Figure 5c shows the plots of RCP transmissitivity and phase provided by the unit cell with variation in orientation angle ($\theta$). The variation in transmittivity can be observed once again with $\theta$, with an average transmittivity of 36% and a variation of 35%. In the same structure, when a 15 nm thin Au sheet is inserted between a nanoparticle and glass substrate, the variation in transmittivity is reduced, along with reduced average transmittivity. The variation in transmittivity is found to be 16%, with an average transmittivity of 13%. Of course, in this case, also there is a big trade-off between the average transmittivity and variation, which necessitates a good choice of dimensions of unit cell and thickness of the metal depending on the application. A better method to fix the dimensions would be to have a full set of design simulations for a particular wavelength, as described in Fig. 2, rather than merely reducing the unit cell size for reducing the quadrupole contribution alone.

The mechanism in any case remains identical. When a thin metallic sheet is inserted between the nanoparticle and the glass substrate, the field localization at the bottom of the nanoparticle is reduced due to charge screening.^29^ As a result, the electric fields are localized around the top corners of the nanoparticle, which suppresses the EQ contribution to the transmittivity with its variations along orientation angles. In addition, the metal layer broadens the dipole resonance, as shown in Fig. 3d. Figure 3d shows the LCP and RCP transmittivity versus wavelength plots for the MoM and MoS unit cell at $\theta = 0^o$. It can be observed that the RCP wavelength response for the MoM unit cell is broader than the RCP wavelength response for the MoS unit cell.

When circularly polarized light interacts with elliptical nanoparticles on a substrate, transverse and longitudinal Electric Dipoles (EDs) along with longitudinal Electric Quadrupoles (EQs), are excited. The transverse ED and longitudinal EQs interact with neighboring particles, causing variations in transmittivity based on the orientation angles due to the changes in interparticle distances. For circularly polarized light, the ED is excited only in an x–y plane along the long axis of the elliptical nanoparticle for the configuration considered. However, EQ is excited along the longitudinal direction (z) and the transverse lobes in an x-y plane along both short and long axis^24,33^ due to the nanoparticle thickness. ED along the y-axis (EDy) is highest and ED along the x-axis (EDx) (Figures involving decomposition of the fields into longitudinal and transverse components are not shown here for brevity) is lowest at $\theta = 0^\circ$, with EDx increasing and EDy decreasing as $\theta$ moves to $90^\circ$ . ED along the z-axis (EDz) remains constant. EQ along the x-z axis (EQxz) peaks at $\theta = 45^\circ$ while EQ along the y-z axis (EQyz) decreases from $\theta = 22.5^\circ$ to $90^\circ$ . Other EQ components are negligible. Overall, EQ is minimized and ED maximized at $\theta = 0^\circ$ and $90^\circ$ , resulting in higher transmittivity, while EQ peaks and ED minimizes at $\theta = 45^\circ$ and $135^\circ$, lowering the transmittivity. As the metal does not support the electric field inside, the longitudinal components of ED and EQ can be suppressed by inserting a metal sheet between the elliptical nanoparticle and the substrate.

While the EQ contribution can be suppressed by reducing nanoparticle dimensions and inter-particle separations, from the point of view of fabrication/experiments, it is desirable to maintain the inter-particle separations to be at least tens of nanometres. Hence, suppression of the components varying with the orientation of nanoparticles is better controlled without reduction in critical dimension, such that the structures are easier to fabricate.

## Experimental demonstration of PM to generate OAM beams and Composite beams in visible spectrum

A vortex phase combined with the phase of the Bessel beam is designed to test PM. At each and every position (*x*, *y*), the local phase will be formed by tunning the orientation angle with the Y-axis of the elliptical nanoparticle. The equation of the geometric phase is given below:

6 $$\begin{aligned} \phi _{G}(x,y)=0.075\sqrt{x^{2}+y^{2}}+\ell tan^{-1}(\frac{y}{x}) \end{aligned}$$

**Figure 6:**
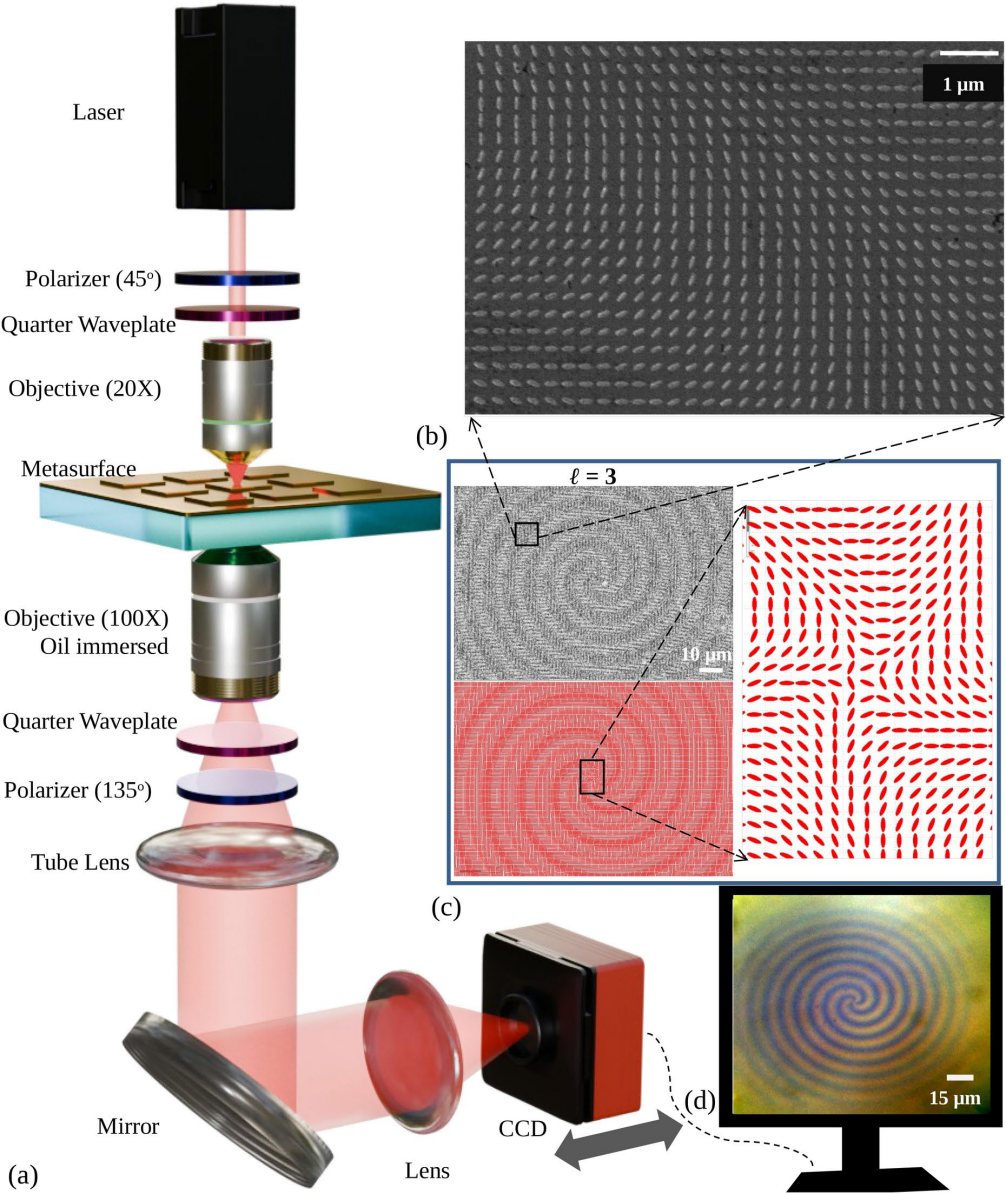
(**a**) Schematic diagram of the experimental setup, (**b**) zoomed SEM image of the fabricated metasurface, (**c**) SEM images of fabricated metasurfaces of $\phi _{G}\mid _{\ell =3}$ in Eq. (6), at the top left, corresponding GDS mask in the bottom left and zoomed GDS mask consist of the unit cell with varied orientation angles at right. (**d**) Broadband zoomed optical image of the metasurface structure with $\phi _{G}\mid _{\ell =3}$ in Eq. (6).

Where $\ell$ is the Topological Charge (TC) of the orbital angular momentum of the vortex phase. The sketch of the experimental setup is depicted in Fig. 6a. A Helium-Neon laser is taken as the input laser source, with a wavelength of 632.8 nm. Light coming from the top is first polarized at an angle of $45^\circ$ using a linear polarizer set at $45^\circ$. After the polarizer, a quarter-wave plate (QWP) is positioned so that its fast axis lines up with the linear polarizer at an angle of $45^\circ$. After passing through the QWP, the light is circularly polarized and directed through a 20X objective lens onto the metasurface. 20X objective helps transform the input light’s spot size comparable to the metasurface size (150 $\upmu$m $\times$ 150 $\upmu$m).

After the metasurface, similarly-handed circularly polarized light has an arbitrary phase, whereas oppositely-handed circularly polarized light contains desired phase information. A 100X immersion objective lens is used to gather both-handed circularly polarized light. After the 100X objective lens, another QWP is added with a fast axis oriented in the orthogonal position of the previous QWP to convert the circularly polarized lights into corresponding linearly polarized light. QWP converts circularly polarized light with the opposite hand to linear polarization at $135^\circ /-45^\circ$, and circularly polarized light with the same hand is converted to $45^\circ$. Another linear polarizer is used, with an orientation of $135^\circ /-45^\circ$, to eliminate the $45^\circ$ polarized light. The light beam is collimated by a tube lens focused on the 100X objective lens’s back focal plane. The light is then directed onto another lens by a mirror. Lastly, a computer-connected, movable Charge-coupled Device (CCD) takes pictures of the output beam. Figure 6b shows the zoomed SEM (secondary electron microscope) images of the fabricated metasurface. Contrast enhanced SEM image of the corresponding metasurface inscribing the phase of $\ell$ = 3; $\phi _{G}\mid _{\ell = 3}$ in Eq. (6) is shown at the top left of Fig. 6c. GDS (graphic design system) of the same metasurface and its zoomed version are shown at the bottom left and right side, respectively, in Fig. 6c. The zoomed optical broadband image of the same metasurface is shown in Fig. 6d. The image is captured to validate the metasurface phase pattern optically after fabrication. The spiral pattern in the image indicates the phase pattern inscribed in the metasurface. The CCD is brought near the lens, and the source is changed to a broadband lamp to capture the image. Samples are characterized using the setup mentioned above in the presence of a red laser (632.8 nm) after fabrication.

In order to demonstrate experimentally our method of flattening the transmittivity, we have chosen different phase patterns that exhibit very high phase gradients on the metasurface. These gradients are chosen so that there are abrupt phase changes leading to intensity nulls at specific points in the beam cross-section. We also show later, through simulations and comparison with the experiment, that if the same patterns had been made without the transmittivity flattening, the observed beams would have been very different.

**Figure 7:**
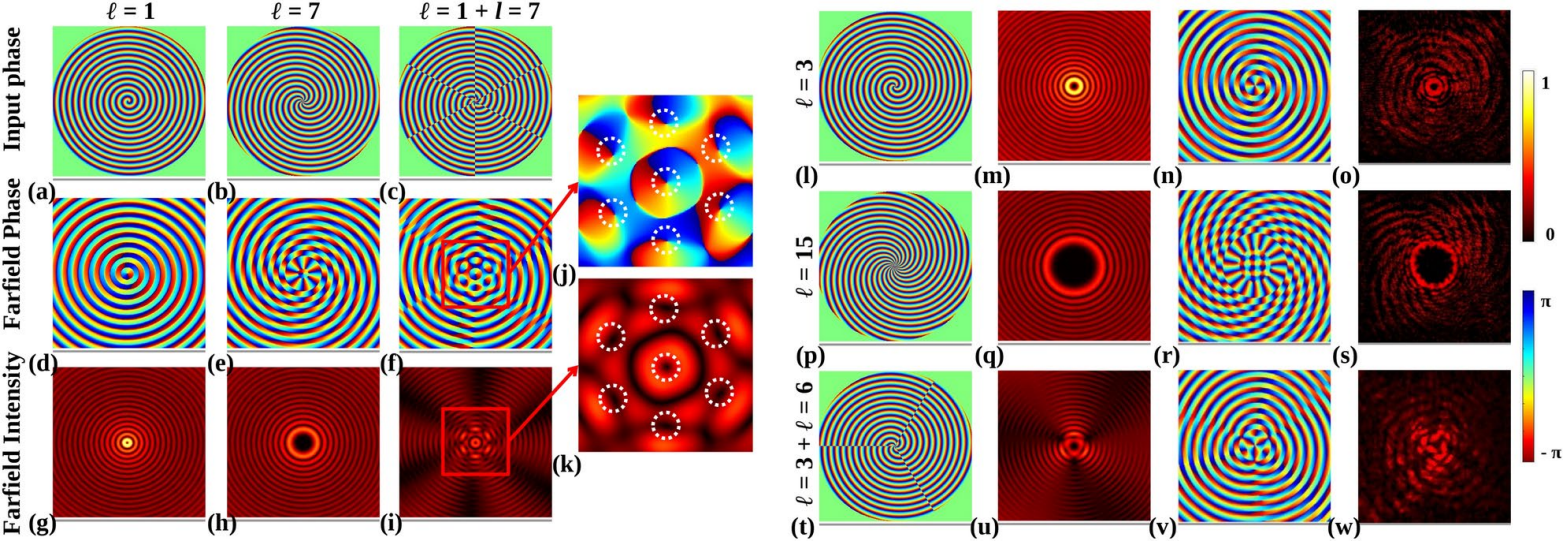
Input phases with (**a**) $\phi _{G}\mid _{\ell =1}$, (**b**) $\phi _{G}\mid _{\ell =7}$ and (**c**) ($\phi _{G}\mid _{\ell =1}+\phi _{G}\mid _{\ell =7}$). (**d**–**f**) Calculated farfield phases of respective input phases, (**g**–**i**) calculated farfield intensities of respective input phases. (**j**) Zoomed farfield phase of (**f**), marked with the red box. (**k**) Zoomed farfield intensity of (**i**), marked with the red box. Each intensity null and phase singularity is marked with a white dotted circle. (**l,p,t**) Input phase of fabricated metasurface: $\phi _{G}\mid _{\ell =3}$, $\phi _{G}\mid _{\ell =15}$, and ($\phi _{G}\mid _{\ell =3}+\phi _{G}\mid _{\ell =6}$) respectively. (**m,q,u**) Respective calculated farfield intensities of input phases. (**n,r,v**) Respective calculated farfield phases of input phases. (**o,s,w**) Respective experimental intensities of input phases.

First, farfield intensities and phase are calculated using the Fourier transform to compare them with experimental intensities. Input phases of $\phi _{G}\mid _{\ell = 1}$, $\phi _{G}\mid _{\ell = 7}$, and ($\phi _{G}\mid _{\ell = 1} + \phi _{G}\mid _{\ell =7}$) are shown in Fig. 7a–c. Corresponding farfield phases are shown in Fig. 7d–f, and farfield intensities in Fig. 7g–i, respectively. Farfield intensity and phase of the third phase are zoomed and shown in Fig. 7k, j, respectively. It is observed that intensity nulls and phase singularities are nicely arranged to form a composite beam. The central intensity null’s size (number of phase rotations) corresponds to the lowest TC of the combination, and the number of intensity nulls (phase singularity) in the outer periphery is the difference between the TCs of the combination.

The independent TC of each intensity null in the outer periphery will always be 1/-1. Here, two combining TCs are 1 and 7. So, the central intensity null corresponds to $\ell$ = 1, and the number of intensity nulls of $\ell$ = 1 each formed at the outer periphery is 6.

Each of the input phases in Eq. (6), $\phi _{G}\mid _{\ell =3}$, $\phi _{G}\mid _{\ell =15}$, and ($\phi _{G}\mid _{\ell =3}+\phi _{G}\mid _{\ell =6}$) is shown in Fig. 7l,p,t. Calculated farfield intensities corresponding to these input phases are shown in Fig. 7m,q,u, and calculated farfield phases are shown in Fig. 7n,r,v. Using the setup depicted in Fig. 6a, finally, the experimental farfield intensities corresponding to the input phases are shown in Fig. 7o,s,w. There is a significant agreement between the calculated and experimental farfield intensities.

The experimental intensity of $\ell$ = 15, as shown in Fig. 7s, shows spiral line intensity in the background instead of circular line intensity, as shown in Fig. 7q. Figure 7w shows the interference intensity between $\phi _{G}\mid _{\ell = 3}$ and $\phi _{G}\mid _{\ell = 6}$. The unique intensity pattern has intensity null in the center and in the periphery.

**Figure 8:**
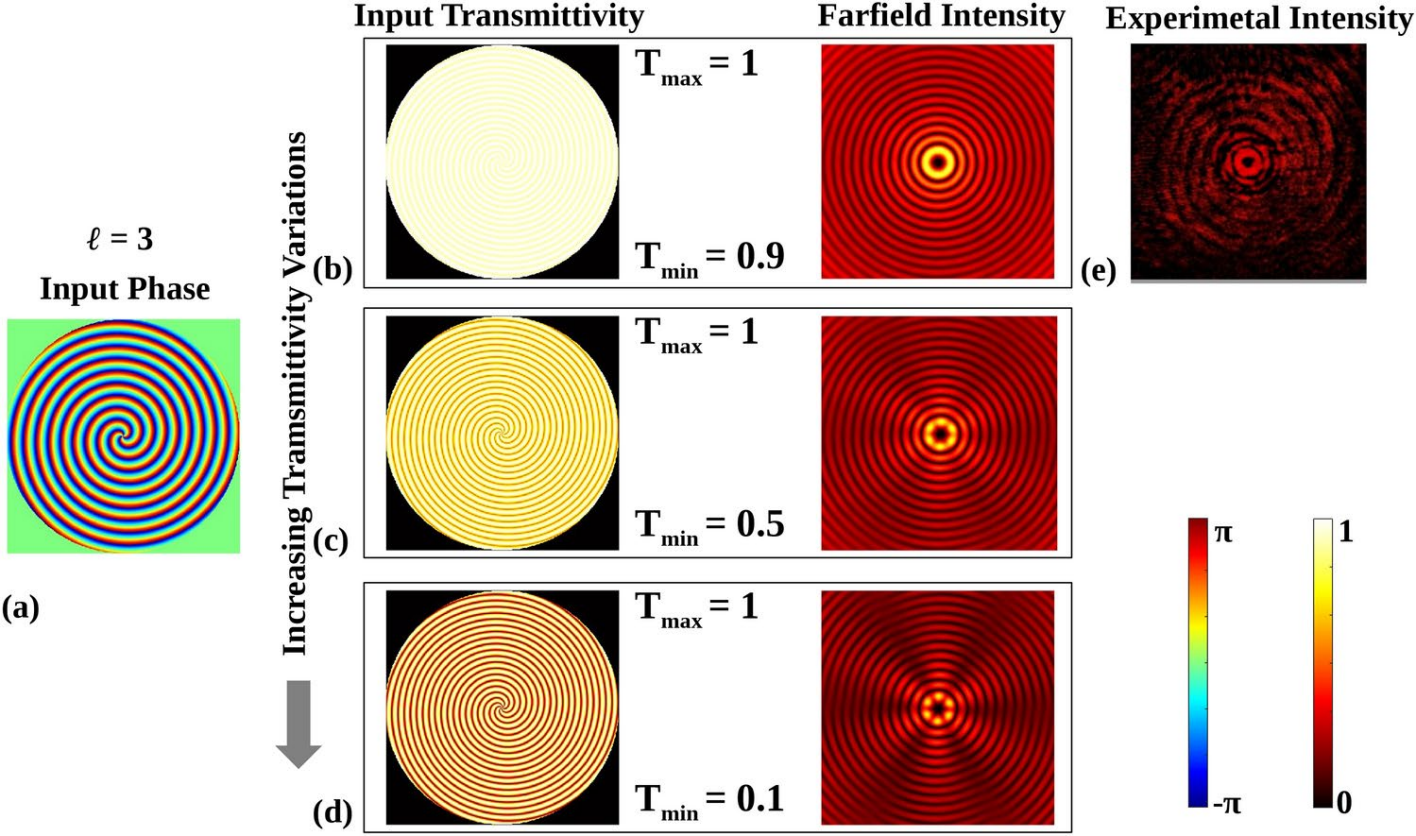
(**a**) Input phase of $\phi _{G}\mid _{\ell =3}$, (**b**) 10% transmittivity variation and corresponding calculated farfield intensity, (**c**) 50% transmittivity variation and corresponding calculated farfield intensity, (**d**) 90% transmittivity variation and corresponding calculated farfield intensity, (**e**) Experimentally obtained farfield intensity.

The number of nulls is interrelated according to the example shown in Fig. 7j,k. The unique intensity pattern is only obtained when two vortex beams of $\ell$ = 3 and $\ell$ = 6 have interfered with each other. Hence, the phase generated by the designed metasurface is validated.

That is because $\ell$ = 15 demands higher phase change, as shown in Fig. 7p, over the unit area near the center, which is not adequately replicated by the metasurface due to the resolution limitation of the unit cell. It is also worth stating that we simulated the phase response and amplitude characteristics of these metasurfaces without the metal sheet, and we noted that through simulations for the variation in transmittivity that would have arisen, these complex beams could not even be achieved. Input phase with a sinusoidal variation of transmittivity mimicking the metasurface without metal sheet according to the response shown in Fig. 3b are taken, and farfield intensities are calculated in Fig. 8.

To analyze the effect of transmission variation on the experimentally generated farfield intensity pattern, a simulation with sinusoidal variation in transmittivity with input taken from Fig. 3b with a phase profile of $\phi _{G}\mid _{\ell =3}$ is presented in Fig. 8. The idea here is to show the effect on the observed vortex beams if the phase metasurface did not have a flat intensity profile. Figure 8a shows the input phase profile mapped to the metasurface. The transmittivity variation profiles and their corresponding simulated farfield intensity profiles for 10%, 50%, and 90% peak to peak change in transmittivity are shown in Fig. 8b–d, respectively. When the variation in transmittivity increases, as indicated by the downward black arrow, the simulated farfield intensity profiles become distorted compared to the ideal intensity profile, which features continuous circular rings. The experimentally obtained farfield intensity pattern using a designed metasurface mapped with the same phase is shown in Fig. 8e. The experimentally obtained intensity profile closely resembles the simulated farfield intensity profile for a 10% variation. Hence, the designed metasurface exhibits complete phase control over reasonably constant transmittivity (nearly 10% transmittivity variation).

## Conclusion

Plasmonic phase metasurfaces suffer from a problem wherein the transmittivities are highly dependent on the orientation of the nanoparticles in a unit cell. By inserting an extremely thin metal sheet between the nanoparticles and the dielectric substrate, we show through simulations and experiments that a constant transmittivity full phase metasurface can be obtained with an elementary Au elliptical nanoparticle in a simple square lattice configuration. We showed that constant transmittivity is achieved by uniform electric dipole moment due to resonance broadening and annihilation of the electric quadrupole moment due to charge screening. Varieties of composite beams were generated experimentally using nanofabricated Metasurfaces to demonstrate the phase control finesse of the designed unit cell. The method also can be extended to different portions of the spectra. Since the resultant fields are concentrated on the top portion of the nanoparticles, there is a significant opportunity to use such structures for surface sensing.

## Methods

### Simulation method

Ansys Lumerical FDTD (Ansys Optics 2022 R2.4, https://www.ansys.com/en-in/products/optics/fdtd) is utilized to perform the simulations. Circularly polarized light is formed by adding two plane waves of orthogonal polarizations with $\pi /2$ phase difference between them. The refractive indices of Au and glass are taken from the software’s library as ‘Au-Johnson and Cristy’ and ‘$\hbox {SiO}_{2}$ -Palik’, respectively. Periodic boundary conditions are applied in positive and negative X and Y directions, and perfectly matched layers are taken in positive and negative Z directions. The minimum mesh size is taken as 0.3 nm during the simulations.

### Fabrication method

A cleaned glass sample is first coated with 2 nm Cr and 15 nm Au using the electron beam evaporation method. Metal-coated samples are spin-coated using polymethyl methacrylate (PMMA) A6 at a 3500 rpm spin speed. Following that, PMMA coated samples are patterned using electron beam lithography. The acceleration voltage is maintained at 30 KV, and the aperture is kept at 10 $\upmu$m during patterning with the dose range of 500–600 $\upmu$ $\hbox {C/cm}^2$. Patterned samples are developed using the MIBK: IPA (1:3) solution. Using Electron beam evaporation, 120 nm of Au is subsequently coated in the samples. Acetone is used for lift-off in order to obtain the final structure.

## Data Availability

The data generated in this paper are available upon reasonable request from the corresponding author.
